# A comparative study of autistic and non-autistic women’s experience of motherhood

**DOI:** 10.1186/s13229-019-0304-2

**Published:** 2020-01-06

**Authors:** A. L. Pohl, S. K. Crockford, M. Blakemore, C. Allison, S. Baron-Cohen

**Affiliations:** 1 Autism Research Centre, Department of Psychiatry, Cambridge University, Douglas House, 18b Trumpington Road, Cambridge, CB2 8AH UK; 2 Autism Asperger Advocacy Australia (A4), Sydney, Australia

**Keywords:** Autism, Motherhood, Parenting

## Abstract

**Background:**

Autism is a lifelong neurodevelopmental difference and disability, yet there is limited research examining parenting in autistic mothers.

**Objective:**

To explore autistic mothers’ experience of the perinatal period and parenthood. This includes pregnancy, childbirth, the postpartum period, self-perception of parenting strengths and weaknesses, communication with professionals in relation to one’s child, mental health difficulties and the social experience of motherhood. It also includes disclosing one’s diagnosis of autism in parenting contexts.

**Methods:**

We used a community-based participatory research model, and recruited an advisory panel, with whom we co-developed an anonymous, online survey for autistic mothers. The online survey was completed by autistic and non-autistic mothers, and we compared their responses using Chi-squared analysis.

**Sample:**

Autistic mothers (*n* = 355), and non-autistic mothers (*n* = 132), each of whom had at least one autistic child, were included in our final analysis.

**Results:**

There were differences in education, gender identity and age of mother at birth of first child. Autistic mothers were more likely to have experienced additional psychiatric conditions, including pre- or post-partum depression, and reported greater difficulties in areas such as multi-tasking, coping with domestic responsibilities and creating social opportunities for their child. They were also more likely to report feeling misunderstood by professionals, and reported greater anxiety, higher rates of selective mutism, and not knowing which details were appropriate to share with professionals. They were also more likely to find motherhood an isolating experience, to worry about others judging their parenting, or feel unable to turn to others for support in parenting. However, despite these challenges, autistic mothers were able to act in the best interest of their child, putting their child’s needs first.

**Conclusions:**

Autistic mothers face unique challenges and the stigma associated with autism may further exacerbate communication difficulties. Greater understanding and acceptance amongst individuals who interact with autistic mothers is needed, and autistic mothers would benefit from additional and better-tailored support.

## Terminology

This article uses identity-first language because autistic individuals have reported a preference for identity-first language (autistic person) rather than person-first language (person with autism) [1].

## Introduction

Autism is a neurodevelopmental condition, diagnosed by difficulties in social-communication alongside a strong preference for repetition, difficulties in adjusting to unexpected change, and a profile of atypical sensory sensitivity. The prevalence of autism is estimated at 1–2% of the population, diagnosed more often in males than females, with a sex ratio of 3:1 (male:female) [2–4]. Little attention has been paid to parenthood in autistic adults, despite autism being a lifelong condition [5]. There are currently no estimates of the number of autistic adults who are parents. Between 17 and 23% of parents of autistic children have the ‘broader autism phenotype’ [6, 7] and autism is partly genetic [8]. Therefore, it is possible that a number of mothers of autistic children may have undiagnosed autism and, because women are on average diagnosed later in life than men [9], some may not receive their diagnosis until they are already parents themselves. Whilst there is some literature on how the presence of an autistic child impacts family dynamics and parents [10, 11], the experience of autistic mothers themselves is relatively unexplored.

To date, only the experience of pregnancy, childbirth and the postpartum period in autistic women have been studied [12, 13]. Using qualitative methods, these studies highlighted themes important to autistic mothers [12, 13]: Heightened sensory experiences during the perinatal period, including breastfeeding; the desire for clear guidance from healthcare professionals and family; stress that follows from the perceived pressure to be a patient and perfect mother; and the stigmatisation of autistic mothers as ‘bad parents’ by healthcare professionals. However, further research of autistic motherhood, beyond the perinatal period, is needed.

The experiences of mothers with intellectual disabilities and psychiatric conditions, which are often co-morbid with autism [14], may inform our understanding of the possible experiences of autistic mothers. Although not all autistic women have an additional diagnosis of a psychiatric condition or intellectual disability, mothers with these diagnoses may face similar challenges to autistic mothers as these conditions are all neurodevelopmental, psychological or behavioural in nature. For women with intellectual disabilities and psychiatric conditions, motherhood is often a desirable experience [15, 16], but for mothers with a psychiatric condition, the stigma associated with their condition has a major impact on how they view themselves as mothers. One study showed they felt as though the concept of the ‘ideal mother’ was incompatible with the negative connotations of their psychiatric condition [15]. Stigma also presents a major barrier to accessing services or seeking support from friends and family. For mothers who fear judgment of their parenting ability and fear potentially losing their child to child protection services, fear of stigmatisation may impede them from accessing services needed by their children. This may be part of a wider feeling of being stigmatised that autistic adults report [17]. Such fears could be well founded as parents with an intellectual disability often face more scrutiny by social services [18] and are at a higher risk of having their parental rights terminated [19].

Autistic individuals are at increased risk of mental health difficulties, compared to neurotypical individuals [20]. It is unclear how this might affect autistic mothers. Feelings of isolation, fear of judgment and the stigma of autism may have an adverse effect on mental health, especially in the early stages of motherhood where women are still adjusting to their new identity [16]. A history of depression is one the highest risk factors for postpartum depression [21]. Given the co-morbidity of depression and autism [20], we would expect autistic mothers to be at higher risk for postpartum depression than are neurotypical mothers, which could isolate them further. This may mean that autistic women require additional tailored support to meet their needs. Awareness of rates of depression in autistic mothers could help support services to anticipate their likely needs, leading to earlier identification of depression symptoms.

Difficulties with processing sensory experiences, for example issues with breastfeeding, and communicating with professionals, such as clinicians, midwives and nurses [12, 13] may be unique components of the motherhood experience for autistic women. Autism is associated with sensory hypersensitivity, often leading to sensory overload [22, 23]. Gardner et al. reported that for some autistic women this made the physical sensation of breastfeeding unpleasant, but that autistic mothers were nevertheless able to successfully breastfeed as they felt it was in the best interest of their child [12]. The ability to overcome difficulties in order to act in the best of interest of the child may play a vital role in the experience of motherhood for autistic women.

The main objective of this study was to provide a preliminary investigation into how autistic women experience the perinatal period and early motherhood, compared to non-autistic mothers. We developed an online survey made widely available to autistic and non-autistic mothers. We wanted to ensure that a wide range of issues were covered and that these issues were truly reflective of the needs of the autistic community. At the time this survey was developed, there was no peer reviewed, published work on autistic mothers. For this reason, we chose to cover a broad range of topics which could inform the priorities for future research, provide some guidance for policy-makers and, most importantly, provide autistic mothers with empirical support when advocating for their individual needs.

## Methods

### Patient and Public Involvement (PPI) model

The research team was approached by the organisation *Autism Women Matter* to conduct a study investigating the experiences of motherhood in autistic women. This study was a collaboration between autistic mothers and academic researchers. Six autistic mothers in the UK were recruited to form an advisory panel. The panel met 4 times and communicated over the telephone or via email with the researchers. The panel contributed to identifying the study objectives and in the design of the survey, conducted through in-person meetings held at the Autism Research Centre, Cambridge, chaired by the lead researchers. The panel was also asked to help disseminate the survey among members of their community and, once the data were collected, was asked to provide input on the interpretation of the qualitative results. Following best PPI practices from the National Institute for Health Research (NIHR) UK, all panel members were reimbursed for their time and travel [24, 25].

### The online survey

The survey asked a range of questions about the experiences of motherhood. These included pregnancy, childbirth and the postpartum period, self-perception of parenting strengths and weaknesses, communication with professionals in relation to one's child, and the social experiences of motherhood, including disclosing one’s diagnosis of autism in parenting contexts.

The survey comprised both forced-choice items, requiring an agree/disagree or yes/no answer, and open-ended questions in which participants were asked to elaborate further. These questions included what influences an autistic woman’s decision to disclose her diagnosis to professionals, and what they find are the positive and negative aspects of motherhood. Using a mixed-methods approach allowed us to gather a broad understanding of the experiences of motherhood; however, the data presented in this study only include the responses to the forced-choice items. Qualitative data from the open-ended questions were not included in this analysis. We also asked questions about any involvement with social services, the results of which are reported in a separate paper (Pohl A, Crockford SK, Blakemore M, Allison C. Baron-Cohen S: “She’s making up his autism”, Autistic mothers’ self-reported experiences with child-protection social services. University of Cambridge. Manuscript submitted for publication; 2019). The panel helped us with the wording of the survey and checked that we used terminology that was both friendly and accessible to the autistic community.

The questions used in the survey were co-created with the panel as we wanted the survey to reflect issues that were specifically pertinent to the autistic mothering community rather than using existing validated measures that may be less relevant to autistic mothers. The aim was to conduct a preliminary study about various aspects of motherhood that might be particularly important to the autistic community. We hoped that this broad approach would highlight future research topics that should be investigated more deeply. The full list of survey items is provided in Additional file 1.

In addition to our survey items, we included a series of comprehensive demographic questions (Table 1), and the short version of the Autism Spectrum Quotient (AQ-10) was administered. The AQ-10 is a measure that quantifies autistic traits in adults [26, 27]. We included this in order to characterise mothers who did not have a clinical autism diagnosis, but who reported being autistic. The AQ-10 uses a 4-point Likert scale scored as binary. In clinical screening, a cut-off is scoring at least 6 or above.

**Table 1:** Demographic characteristics of autistic mothers and non-autistic mothers

	Autistic	Non-autistic	*p value*
Age (years)	42.7 ± 8.4	44.6 ± 9.1	0.041
Sex (%)			
Female	352 (99%)	132 (100%)	0.947
Male	2 (<1%)	-	
Gender (%)			
Female	339 (95%)	129 (98%)	0.018
Male	2 (<1%)	3 (2%)	
Other	14 (4%)	-	
Education			
Some secondary/high school	20(6%)	9 (7%)	0.011
Completed secondary/high school	55 (15%)	25 (19%)	
Completed some of an undergraduate degree	71 (20%)	10 (8%)	
Completed an undergraduate degree	102 (28%)	35 (26%)	
Completed a postgraduate/graduate degree	107 (30%)	53 (40%)	
Marital status			
Single	36 (10%)	12 (9%)	0.033
Married	202 (57%)	88 (67%)	
Civil partnership	9 (2.5%)	1 (<1%)	
Divorced	44 (12%)	3 (2%)	
Widowed	4 (1%)	2 (1.5%)	
Separated	28 (8%)	9 (7%)	
Long-term partner	29 (8%)	16 (12%)	
Other	3 (<1%)	1 (<1%)	
Living with current partner (if applicable)			
Yes	240 (99%)	103 (98%)	1
No	3 (1%)	2 (2%)	
Does partner have autism? (if applicable)			
Diagnosed	16 (6%)	2 (2%)	0.089
Suspected	75 (31%)	27 (26%)	
No	152 (63%)	76 (72%)	
Single parenthood			
Currently a single parent	98 (28%)	23 (17%)	0.003
Have been a single parent in the past	79 (22%)	20 (15%)	
Never have been a single parent	178 (50%)	89 (67%)	
Age at first birth (years)	27.7 ± 6.0	29.5 ± 6.0	0.002
Number of children (median)	2.4	2.4	0.701
Age of first child (years)	15.2 ± 8.5	15.3 ± 8.9	0.891
Average age of all children (years)	12.9 ± 8.0	12.9 ± 7.5	0.932
Country (top 5 shown)			
United Kingdom	173 (48%)	75 (57%)	0.314
United States	80 (22.5%)	23 (17%)	
Australia	45 (13%)	23 (17%)	
France	11 (3%)	4 (3%)	
Canada	12 (3%)	2 (1.5%)	
Proportion accounted for by top 5 countries	321 (89.5%)	127 (94.5%)	

The survey was made available online and disseminated via autism-specific support groups and social media pages. All responses were anonymous to preserve participant confidentiality. Ethical approval was obtained from the Psychology Research Ethics Committee at the University of Cambridge. Additional approval was obtained from Her Majesty’s Court and Tribunal Services (HMCTS) to ensure no legal action could be taken against participants who might disclose details from closed care proceedings. The survey can be found in Additional file 1. Data from the survey were analysed using R (Version 1.1.383 – © 2009-2017 RStudio, Inc).

### Participants

Participants included autistic and non-autistic mothers who were parents, regardless of whether they were the biological parent of the child. Recruitment was targeted towards mothers who had at least one autistic child, of any age. This was to ensure that any differences in results could be attributed to a mother being autistic, rather than having an autistic child. 410 autistic mothers and 258 mothers without a diagnosis of autism (henceforth non-autistic mothers) completed the survey. After matching the groups for having at least one diagnosed and/or suspected autistic child, this reduced the sample of non-autistic mothers to 132 and the sample of autistic mothers to 355. Five per cent of non-autistic mothers and 2% of autistic mothers were not the biological mother of their child. The sample mainly comprised mothers from Western countries (Table 1); other countries included Sweden (*n* = 3), New Zealand (*n* = 3), Israel (*n* = 1), Greece (*n* = 2), South Africa (*n* = 1), Serbia (*n* = 1), Belgium (*n* = 2), Italy (*n* = 1), UAE (*n* = 1), Denmark (*n* = 5), Costa Rica (*n* = 1), Brazil (*n* = 1), Monaco (*n* = 1), Mexico (*n* = 2), Ireland (*n* = 5), Netherlands (*n* = 4), and Germany (*n* = 3). See Table 1 for demographic characteristics.

Approximately two-thirds of our sample of autistic mothers reported having a diagnosis of autism, while the remaining third reported self-identified as autistic but did not have a clinical diagnosis. We included these mothers in the autistic group because, although the diagnosed mothers averaged a higher score on the AQ-10 (diagnosed: mean = 7.4 (sd = 1.4), self-identified: mean = 6.4 (sd = 1.8), *df*=186.76, *p* < 0.001), both diagnosed and self-identified autistic mothers scored significantly higher than non-autistic mothers (*p* < 0.001) (Table 2) and scored above the cut-off of 6 or more. Furthermore, including the self-identified group ensured we did not exclude anyone’s autism experience. Therefore, for the subsequent analyses, we collapsed these two groups, into one group of autistic mothers. Within this group, over 60% of autistic mothers were diagnosed or became aware of their own autism following their child receiving a diagnosis; 6% of the self-identified mothers had experienced refusal by clinicians to give them an autism diagnosis (Table 3). Finally, autistic mothers were more likely to have a self-reported additional psychiatric and/or psychological diagnosis (*χ*^2^ = 8.392, *df* = 1, *p* = 0.001) compared to the non-autistic mothers.

**Table 2:** AQ-10 scores by diagnostic status

	Non-autistic	Autistic		
		Diagnosed	Self-identified	Combined
*N*	132	235	120	355
		66.2%	33.8%	
AQ-10 ± SD	3 ± 1.6	7.4 ± 1.4	6.4 ± 1.8	7.08±1.6

**Table 3:** Further information on psychiatric diagnostic profile

	Non-autistic	Autistic		*p value*
		Diagnosed	Self-identified	
Received an autism diagnosis or suspected they were autistic following the birth of one of their children				
Yes	-	142 (61%)	88 (73%)	0.007
No		66 (28%)	16 (13%)	
Not applicable		26 (11%)	16 (13%)	
Had a diagnosis of autism refused				
Yes	-	-	7 (6%)	0.016
No	131 (99%)	-	113 (94%)	
Not applicable	1 (<1%)	235 (100%)	-	
Has a diagnosis of another psychiatric/psychological condition				
Yes	59 (45%)	171 (72%)	92 (77%)	0.004
No	73 (55%)	64 (27%)	28 (23%)	

## Results

### The experience of pregnancy and early infancy

Autistic mothers were significantly more likely than non-autistic mothers to experience both prenatal depression (*χ*^2^ = 9.534, *df* = 2, *p* = 0.009) and postnatal depression (*χ*^2^ = 10.401, *df* = 2, *p* = 0.006). There was also a significant difference between groups in reporting whether they had the process of birth explained, with autistic mothers more likely to feel like birth had not been adequately explained to them, in comparison with non-autistic mothers (*χ*^2^ = 14.597, *df* = 2, *p* = 0.0007). However, there was no significant difference between groups in antenatal class attendance (*χ*^2^ = 0.965, *df* = 2, *p* = 0.617); see Table 4.

**Table 4:** Experiences of pregnancy

	Autistic	Non-autistic	*p value*
Experienced antenatal depression			
*N*	355	132	0.008
Yes	141 (40%)	33 (25%)	
No	210 (59%)	96 (72%)	
Not applicable	4 (1%)	3 (2%)	
Attended antenatal classes			
*N*	355	132	0.617
Yes	239 (67%)	95 (72%)	
No	113 (32%)	36 (27%)	
Not applicable	3 (1%)	1 (<1%)	
Had the process of birth explained			
*N*	355	132	0.001
Yes	219 (62%)	105 (79%)	
No	121 (34%)	22 (17%)	
Not applicable	15 (4%)	5 (4%)	
Experienced postpartum depression			
*N*	354	132	0.012
Yes	213 (60%)	59 (45%)	
No	137 (39%)	69 (52%)	
Not applicable	4 (1%)	4 (3%)	

Difficulties with breastfeeding were an issue specifically raised by our panellists, in particular with issues in producing adequate milk supply. There was no significant difference between groups in whether mothers attempted to breastfeed their first or second child (Table 5). Mothers were asked to answer questions about breastfeeding each child they reported having. On average mothers reported having two children and over 70% of mothers in both groups had two or less children, so only data given about the first two children are reported here. There were no significant differences between whether the mothers had a low milk supply for their first and second child and whether they had any difficulties breastfeeding their first child (Table 5). However, significantly more autistic mothers reported having difficulties breastfeeding their second child than non-autistic mothers (*χ*^2^ = 4.934, *df* = 1, *p* = 0.026).

**Table 5:** Experience of breastfeeding

	Autistic	Non-autistic	*p value*
Attempted to breastfeed their 1st child			
*N*	355	132	0.712
Yes	314 (88%)	119 (90%)	
No	41 (12%)	13 (10%)	
Attempted to breastfeed their 2nd child			
*N*	268	107	0.536
Yes	219 (82%)	91 (85%)	
No	49 (18%)	16 (15%)	
Had low milk supply for 1st child			
*N*	310	119	0.155
Agree	115 (37%)	42 (35%)	
Disagree	195 (63%)	77 (65%)	
Had low milk supply for 2nd child			
*N*	218	91	0.155
Agree	67 (31%)	20 (22%)	
Disagree	151 (69%)	71 (78%)	
Did not have difficulties breastfeeding their 1st child			
*N*	306	118	0.375
Agree	111 (36%)	49 (42%)	
Disagree	195 (64%)	69 (58%)	
Did not have difficulties breastfeeding their 2nd child			
*N*	217	89	0.026
Agree	107 (49%)	57 (64%)	
Disagree	110 (51%)	32 (36%)	

### Differences in parenting styles

Autistic mothers reported more difficulty than non-autistic mothers with the multi-tasking demands of parenting (*χ*^2^ = 67.823, *df* = 1, *p* < 0.0001), with the domestic responsibilities (*χ*^2^ = 54.279, *df* = 1, *p* < 0.0001), creating socialising opportunities for their child (*χ*^2^ = 23.239, *df* = 1, *p* < 0.0001), and were less likely than non-autistic mothers to perceive themselves as organised parents (*χ*^2^ = 21.208, *df* = 1, *p* < 0.0001). There was no difference between autistic and non-autistic mothers’ ability to prioritise their child’s needs above their own (*χ*^2^ = 0.0042, *df* = 2, *p* = 0.948), or in seeking opportunities to boost their child’s self-confidence (*χ*^2^ = 1.621, *df* = 1, *p* = 0.203) (Table 6).

**Table 6:** Experience of parenthood

	Autistic	Non-autistic	*p value*
I am an organised parent			
*N*	355	131	0.0001
Agree	199 (56%)	104 (79%)	
Disagree	156 (44%)	27 (21%)	
I prioritise my child's needs above my own			
*N*	354	132	0.948
Agree	341 (96%)	128 (97%)	
Disagree	13 (4%)	4 (3%)	
I can cope with the multi-tasking that parenting requires			
*N*	355	131	0.0001
Agree	188 (51%)	123 (94%)	
Disagree	167 (49%)	8 (6%)	
I can cope with all the domestic responsibilities of parenthood			
*N*	355	132	0.0001
Agree	167 (47%)	112 (85%)	
Disagree	187 (53%)	20 (15%)	
I look for opportunities to boost my child’s self-confidence			
*N*	355	131	0.203
Agree	339 (95%)	129 (98%)	
Disagree	16 (5%)	2 (2%)	
I put effort into trying to create opportunities for my child to socialise			
*N*	355	132	0.0001
Agree	251 (71%)	121 (92%)	
Disagree	104 (29%)	11 (8%)	

Within the group of autistic mothers, the majority (61%) felt they should be offered extra support because of their diagnosis, and 41% of mothers felt that the support they received from agencies was inadequate, compared to 14% who believed it to be adequate (Table 7).

**Table 7:** Support needs specific to autistic mothers

	Agree	Disagree	Not applicable
I feel I should be offered extra support in my parenting, due to my autism spectrum condition			
*N*	213	135	
	61%	39%	-
When I have asked for extra support from agencies in order to meet my needs as a parent, I have received the support I required			
*N*	48	146	157
	14%	41%	45%

### Communicating with child professionals

Professionals was used as the general term to refer to clinicians, teachers, paediatricians or social workers; anyone whom a mother might come into to contact with when advocating on behalf of her child. Autistic mothers reported significantly more difficulty than non-autistic mothers in being able to communicate effectively with professionals about their child (*χ*^2^ = 32.674, *df* = 1, *p* = 0.0001) and experienced a greater amount of anxiety when needing to interact with professionals (*χ*^2^ = 44.411, *df* = 1, *p* = 0.0001). Forty-four per cent of autistic mothers reported that this caused them to experience difficulties with communication (*χ*^2^ = 61.023, *df* = 1, *p* = 0.0001). In comparison with non-autistic mothers, autistic mothers were also more likely to feel misunderstood by professionals (*χ*^2^ = 19.281, *df* = 1, *p* = 0.0001) and to end up in conflict over their child with professionals (*χ*^2^ = 6.888, *df* = 1, *p* = 0.009). Finally, autistic mothers struggled more than non-autistic mothers to know which personal details are most appropriate to share with professionals (*χ*^2^ = 63.637, *df* = 1, *p* = 0.0001). A large majority (70%) of autistic mothers reported being able to communicate well with professionals, while 60% of those mothers also reported experiencing such high levels of anxiety that they could not think clearly (Table 8).

**Table 8:** Experience of advocating on behalf of their child

	Autism	Non-autistic	*p value*
I communicate well with professionals about my child			
*N*	354	131	0.0001
Agree	249 (70%)	125 (95%)	
Disagree	105 (30%)	6 (5%)	
I often end up in conflict with professionals about my child			
*N*	353	131	0.009
Agree	159 (45%)	41 (31%)	
Disagree	194 (55%)	90 (69%)	
I find that professionals involved with my child often don't believe me			
*N*	348	131	0.0001
Agree	189 (54%)	41 (31%)	
Disagree	159 (45%)	90 (69%)	
I find it easy to know which details about my child and family are appropriate to share with professionals involved with my child			
*N*	351	131	0.0001
Agree	150 (43%)	110 (84%)	
Disagree	201 (57%)	21 (16%)	
I find talking to professionals about my child causes me so much anxiety that I am unable to think clearly			
*N*	351	131	0.0001
Agree	210 (60%)	33 (25%)	
Disagree	141 (40%)	98 (75%)	
I find talking to professionals about my child causes me so much anxiety that I experience forms of communication difficulty			
*N*	352	132	0.0001
Agree	156 (44%)	8 (6%)	
Disagree	196 (56%)	124 (94%)	

### Disclosing an autism diagnosis to professionals

We compared the responses for groups of mothers with a clinical autism diagnosis to those who self-identify as autistic, as both groups may still face similar difficulties when deciding to disclose their autism. More diagnosed mothers disclosed their autism than self-diagnosed mothers (*χ*^2^ = 70.703, *df* = 4, *p* = 0.0001). However, the majority of autistic mothers with a diagnosis still reported disclosing their diagnosis ‘sometimes’, ‘rarely’ or ‘never’, suggesting that even with a diagnosis, autistic mothers are still reluctant to disclose to professionals (Table 9). We also found that regardless of having a clinical diagnosis or not, over 80% of autistic mothers worried that once they disclosed their autism to a professional, a professional’s attitude towards them would change (*χ*^2^ = 0.966, *df* = 1, *p* = 0.326). No differences were found between diagnosed and non-diagnosed autistic mothers on whether they had ever encountered disbelief regarding their diagnosis (*χ*^2^ = 9.405, *df* = 4, *p* = 0.052). Regardless of having the validation of a clinical diagnosis, both groups reported encountering disbelief the majority of the time once they disclosed their autism to a professional (Table 9).

**Table 9:** Experience of autism disclosure

	Autism		
	Diagnosed	No diagnosis	*p value*
How often do you disclose your autism spectrum condition to professionals when talking about your child?			
*N*	231	117	0.0001
All the time	43 (18%)	8 (7%)	
Often	43 (18%)	2 (2%)	
Sometimes	57 (25%)	20 (17%)	
Rarely	58 (25%)	27 (23%)	
Never	30 (13%)	60 (51%)	
If I were to disclose my autism to a professional, I would worry about whether this person’s attitude towards me would change as a result of disclosure			
*N*	234	115	0.326
Agree	204 (87%)	95 (83%)	
Disagree	30 (13%)	20 (17%)	
Do you ever encounter disbelief about your autism after disclosing this diagnosis to a professional?			
*N*	226	94	0.052
All the time	45 (20%)	14 (15%)	
Often	62 (27%)	26 (27%)	
Sometimes	30 (13%)	16 (17%)	
Rarely	24 (10%)	20 (21%)	
Never	65 (29%)	18 (19%)	

### Personal experiences of motherhood

Autistic mothers were significantly more likely to find motherhood an isolating experience (*χ*^2^ = 4.37, *df* = 1, *p* = 0.037), to feel as though their parenting was being judged (*χ*^2^ = 27.293, *df* = 1, *p* = 0.0001) and to feel unable to ask for support, even when they felt they needed it (*χ*^2^ = 27.247, *df* = 1, *p* = 0.0001). Autistic mothers were also more likely to report they were not coping (*χ*^2^ = 18.623, *df* = 1, *p* = 0.0001) and less likely to find motherhood a rewarding experience than non-autistic mothers (*χ*^2^ = 4.67, *df* = 1, *p* = 0.031), although it is important to note that 85% of autistic mothers did indeed report that motherhood was rewarding to them (Table 10).

**Table 10:** Overall experience of motherhood

	Autism	Non-autistic	*p value*
I have found motherhood to be a rewarding experience			
*N*	352	132	0.031
Agree	304 (86%)	124 (94%)	
Disagree	48 (14%)	8 (6%)	
I have found motherhood to be an isolating experience			
*N*	350	130	0.036
Agree	242 (69%)	76 (58%)	
Disagree	108 (31%)	54 (42%)	
I am not afraid of others judging my parenting			
*N*	352	132	0.0001
Agree	129 (37%)	84 (64%)	
Disagree	223 (63%)	48 (36%)	
I am able to turn to others for support in parenting when I need it			
*N*	351	132	0.0001
Agree	144 (41%)	90 (68%)	
Disagree	207 (59%)	42 (32%)	
I have often felt I am not coping in being a mother			
*N*	354	132	0.0001
Agree	241 (68%)	61 (46%)	
Disagree	113 (32%)	71 (54%)	

## Discussion

Motherhood in autistic women is a neglected area in autism research. Our findings demonstrate that there are aspects of parenthood which autistic mothers find more difficult than non-autistic mothers (who do not have a formal diagnosis of autism or self-identify as autistic, but who have an autistic child). Critically, these included difficulties in communicating with professionals, negative perceptions of their mothering, such as fear of judgement of their parenting skills by others, and high rates of postpartum depression. In addition, there are challenges unique to being an autistic parent, such as deciding when not to disclose their autism. We also identified positive aspects of motherhood for autistic women and that, for an overwhelming majority of autistic mothers, parenting was overall a rewarding experience. It is important to note that there were statistically significant differences between our groups with regard to some of their demographic characteristics, such as age, marital and educational status and average age at first birth. Future studies should aim to match groups on these variables to examine whether these influence the results.

Autistic mothers reported more difficulties interacting with professionals, such as clinicians or social workers throughout their experience of parenting. More non-autistic than autistic mothers felt they had the process of birth explained to them in a way they could understand. Our findings highlight how autistic mothers may be more susceptible to difficulties communicating and interacting with professionals during their pregnancy [13]. Autistic mothers also reported that they were reluctant to disclose they had autism. Indeed, over 80% of mothers worried that disclosing their autism would affect a professional’s attitude towards them and nearly 40% of mothers with a diagnosis reported that they rarely or never disclosed. For mothers who suspected they were autistic but did not have a diagnosis, this increased to 75%. Previous research has shown how perceived stigma of one’s diagnosis of a disability or mental health condition can affect one’s perception of motherhood [14]. Autistic mothers in our sample reported feeling like motherhood was a more isolating experience than non-autistic mothers and felt as though they were being judged on their parenting skills, a theme also reported by Rogers and colleagues [13]. Autistic mothers were more likely to feel they were not coping as parents and to feel they were unable to turn to others for support. In addition, autistic mothers may fear this negative perception in professionals, such as clinicians or social workers, leading to a fear or unwillingness to disclose their autism.

Fear of judgement from others may be linked to interaction difficulties, where over 40% of autistic mothers found speaking to professionals was so anxiety inducing they were either unable to think clearly, or experienced difficulties in communication. Furthermore, perceived stigma and fear of being viewed as a ‘bad parent’ might deter autistic mothers from asking for much needed tailored support. If autistic mothers are less likely to approach other parents or professionals for advice and emotional support, this could create a vicious cycle whereby parenting difficulties may become overwhelming, leading, for example, to feelings of isolation. Our findings highlight the emotional toll motherhood may take on autistic women, which could be further exacerbated by lack of awareness and acceptance, and tailored support services. Therefore, it is important to ensure that there is a broader understanding of the challenges associated with being an autistic mother among professionals. By furthering professionals’ understanding and awareness, this will hopefully decrease stigma associated with autism, which may be preventing autistic mothers from disclosing their diagnosis. We also hope that it will help ensure that autistic mothers are able to receive the support they require and effectively advocate for their children.

Research on the experiences of pregnancy and early infancy for autistic mothers has highlighted challenges that may be associated with sensory processing and difficulties [12, 13]. However, despite these sensory issues, most mothers in our sample were able to successfully breastfeed their child, with over 80% of autistic mothers attempting to breastfeed their first two children. It may be that autistic mothers were able to override any unpleasant tactile sensations associated with breast feeding in order to do what they believed was best for their child, and this hypothesis needs to be formally tested in the future.

There were also no significant differences in the proportion of autistic and non-autistic mothers who had difficulties breastfeeding their first child, although an increased number of autistic mothers reported having difficulties with their second child. It is possible that the tactile unpleasantness of breastfeeding proves too much for autistic women, that by the second child they find it much more difficult to bear. However, it is also reasonable to argue that given the cumulative average age of reported children in our study was 12 years, autistic mothers in our sample had a better recall of their experience breastfeeding their second child in comparison with their first. If this is true, it would support previous findings that tactile sensations, such as breastfeeding, are unpleasant to autistic mothers, given the heightened processing of sensory information in autism [28]. Further research is necessary to better understand the relationship between autism and breastfeeding. As others have also reported that autistic mothers have a high degree of interest in the benefits of breastfeeding and engagement with breastfeeding [12], lactation consultants and breastfeeding support organisations such as La Leche League might be a key professional group that could benefit from further training about interacting with autistic mothers.

We also asked mothers about their lifelong experiences of parenting, which goes beyond previous research [12, 13, 29] focusing mainly on the early stages of motherhood. Consistent with findings of executive function difficulties in autism, which include poorer performances on measures of planning and mental flexibility than neurotypical adults [28], autistic mothers reported greater difficulty with multi-tasking, organisation and domestic responsibilities. Difficulties with social-communication and planning, organising, multi-tasking, and a strong need for routine, may be exacerbated when autistic individuals are caring for their family. In a follow-up question on parenting needs, 62% autistic mothers felt that they needed extra support because of their autism. Although executive function has been extensively researched [30, 31], how difficulties in these domains may influence autistic parenting skills is unknown. Translating interventions targeted at mitigating executive function difficulties in adult life to the specific responsibilities of parenthood may benefit the autistic parenting community.

In terms of positive outcomes, 96% autistic mothers were able to prioritise their child’s needs above their own and seek ways in which they can boost their child’s self-confidence. Findings such as these highlight how, despite the challenges with managing everyday domestic life, autistic mothers can overcome these in order to care for their child. This was further supported by 86% of autistic mothers who reported they found parenthood rewarding. Similar to the results about breastfeeding, autistic mothers were able to overcome challenges unique to their autism, such as executive function difficulties and sensory issues, to act in the best interest of their child. Although we found a slight decrease in efforts by autistic mothers to provide opportunities for their child to socialise (which could be due to having to socialise themselves with other mothers and/or parents) 73% of autistic mothers still reported that they were capable of doing so.

In addition to autism, over 70% of mothers, both with and without a formal diagnosis of autism, reported having an additional psychiatric condition, in comparison with only 41% of our non-autistic sample. Autistic mothers also reported being more likely to suffer from both prenatal and postnatal depression, with nearly 60% reporting having experienced postpartum depression. Autistic individuals were four times more likely to experience depression [19, 32] and have higher co-morbidity rates with other conditions such as anxiety and personality disorders [33]. Given that autistic individuals rated improvements in mental health interventions as a top priority for autism research [34], our findings highlight how more research is needed to understand the implications of postpartum depression for autistic women.

Whilst the high rates of mental health conditions in our sample may reflect a wider issue among the autistic population, postpartum depression is linked exclusively with motherhood. Postpartum depression can have serious consequences for both mother and child, but there are effective treatments for postpartum depression and screening tools to identify those that would benefit [35, 36]. However, given that autistic mothers may withstand higher scrutiny from social services and medical professionals, be more likely to have their parental rights terminated resulting in the loss of their child [19] and fear that their parenting abilities are overall constantly being criticised and judged [13], acknowledging postpartum depression and, in turn, seeking treatment may not feel like a viable option. Our study shows a higher rate of postpartum depression in autistic mothers than in non-autistic mothers. However, we did not employ a validated measure of postpartum depression or follow-up our questions with further details on the condition. Developing appropriate screening tools and successful interventions that specifically target postpartum depression in autistic mothers should be a new research priority. Untangling the relationships between depression, psychosocial stressors and autism is a pressing issue for autistic mothers.

We stress the importance of using a PPI model in research with autistic individuals. Themes central to autistic mothers were initially brought to our attention by the panel, and those have now also appeared in studies of motherhood in autism [12, 13, 27]. By listening to the autistic community and collaborating with them to design our research, we were able to design a study that was informed by and representative of autistic mothers, with themes about motherhood that are relevant to them.

### Limitations

To our knowledge, this is the first study to address the experience of motherhood in autistic women beyond the perinatal period. Our survey did not always explore context-specific issues. Therefore, the data reported here should be viewed as exploratory. We hope that this will provide the foundations for future research and will help autistic mothers to obtain the support they require.

Our non-autistic sample may not be representative of the general population of mothers. Our non-autistic sample only included mothers with at least one autistic child and included a higher than usual proportion of women who had experienced postpartum depression. Whilst average rates in population samples are 10–15% [37, 38], 45% of non-autistic mothers in our sample reported experiencing postnatal depression following the birth of at least one of their children. Additionally, mothers in our samples were also from predominantly Western countries, suggesting that the themes reported here may not be applicable to women from non-Western countries. Finally, 6% of mothers who reported self-identifying as autistic were not given an autism diagnosis by a clinician. This reflects that our sample of women may not be representative of both the general and autistic population of mothers and therefore may reduce the generalisability of our findings.

Furthermore, given the nature of the study, only mothers who were literate, able to understand our questions and with access to a computer were able to complete the survey, again highlighting that the results from this survey may not be representative of all autistic mothers in the population. We also deliberately chose to compare autistic mothers with mothers who were not autistic but who had an autistic child, which allowed us to control for the potential additional stress of having an autistic child. We referred to the non-autistic group throughout this paper as ‘non-autistic mothers’ rather than ‘neurotypical mothers’ because for genetic reasons we should assume that this group included a significant proportion of mothers with the ‘broader autism phenotype’ [7], although the average AQ-10 scores were still within the neurotypical range. Future studies should include a neurotypical non-autistic group. We would predict that there will be significant differences between autistic mothers and a representative sample of non-autistic mothers.

Finally, the average of age of children and mothers in our study was quite high, with children being adolescents and mothers about 40 years of age at the time of completion of the survey. Responses may therefore be influenced by recall bias, whereby mothers were asked to recollect experiences, e.g. breastfeeding, that may have happened over a decade prior to taking part in this study. Overall, it is very likely our results are not generalisable to all autistic mothers and do not represent the whole spectrum of experiences, difficulties or issues specific to autistic mothers. However, we hope that this preliminary investigation into the differences in experiences between autistic and non-autistic mothers will provide a platform for discussion and help direct future research.

## Conclusions

There is a need for both increased awareness and acceptance of the experiences of motherhood for autistic women and the need for more tailored support services. Many issues that we identified could be attributed to perceived stigma of autism, lack of awareness and unmet support. Communication difficulties with professionals, feelings of isolation and perceived judgment may create further barriers for autistic mothers to ask for the support they need. Autistic mothers also showed a higher rate of mental health difficulties, with a very high rate (58%) of autistic mothers reporting having suffered from postpartum depression. However, this study also demonstrates that autistic mothers are highly resilient and able to overcome their difficulties to put their child’s needs first. Further research should explore the experience of parenting for autistic individuals and ensure that these findings are used to significantly improve the everyday life of autistic mothers and fathers.

## Supplementary information

**Additional file 1.** Survey on Motherhood Experiences (excluding demographic questions).

## Data Availability

The datasets generated during and/or analysed during the current study are not publicly available due to the sensitive nature of some of the data, but are available from the corresponding author on request, and such requests will be considered by the authors.

## Abbreviations

UK: United Kingdom
PPI: Patient and Public Involvement
AQ-10: Autism Quotient—10 items
UAE: United Arab Emirates
sd: standard deviation
df: degrees of freedom
