# Supplementary material for: A comparative study of autistic and non-autistic women’s experience of motherhood
Source: Mol Autism. 2020 Jan 6;11:3. doi: 10.1186/s13229-019-0304-2 (PMC6945630; doi:10.1186/s13229-019-0304-2)
Supplement: Supplementary file 1 — Additional file 1. Survey on Motherhood Experiences (excluding demographic questions). [file 13229_2019_304_MOESM1_ESM.docx]

**Additional file 1**

**Survey on Motherhood Experiences (excluding demographic questions)**

**Pregnancy, Childbirth, Breastfeeding, and Infancy**

Did you experience antenatal (prenatal) depression prior to the birth of any of your children?

- Yes
- No
- Not Applicable

Did you attend antenatal (prenatal) classes prior to the birth of your first child?

- Yes
- No
- Not Applicable

Answer If No Is Selected Or Not Applicable Is Selected

If you did not attend antenatal (prenatal) classes, could you briefly explain why you chose not to attend?

|  |
| --- |

The process of labour and delivery (or what is involved in a C-section) was explained to me in a way I could understand when I delivered my first child.

- Strongly Agree
- Agree
- Disagree
- Strongly Disagree
- Not Applicable

Did you experience post-natal depression following the birth of any of your children?

- Yes
- No
- Not Applicable

*This section also loops based on number of children.*

Did you breastfeed or attempt to breastfeed your first/second/third/etc. child?

- Yes
- No

Answer If Yes Is Selected

I had a low milk supply while breastfeeding my first/second/third/etc. child.

- Strongly Agree
- Agree
- Disagree
- Strongly Disagree

Answer If Yes Is Selected

I did not have any difficulties breastfeeding my first/second/third/etc. child.

- Strongly Agree
- Agree
- Disagree
- Strongly Disagree

Answer If Disagree Is Selected Or Strongly Disagree Is Selected

What sort of difficulties did you have breastfeeding your first/second/third/etc. child?

|  |
| --- |

**Parenting Styles**

I am an organized parent.

- Strongly Agree
- Agree
- Disagree
- Strongly Disagree

I prioritize my child's needs above my own.

- Strongly Agree
- Agree
- Disagree
- Strongly Disagree

I can cope with the multi-tasking that parenting requires.

- Strongly Agree
- Agree
- Disagree
- Strongly Disagree

I can cope with all the domestic responsibilities of parenthood.

- Strongly Agree
- Agree
- Disagree
- Strongly Disagree

I look for opportunities to boost my child's self confidence.

- Strongly Agree
- Agree
- Disagree
- Strongly Disagree

I put effort into trying to create opportunities for my child to socialize.

- Strongly Agree
- Agree
- Disagree
- Strongly Disagree

**Social interactions about my child**

I communicate well with professionals about my child.

- Strongly Agree
- Agree
- Disagree
- Strongly Disagree

I often end up in conflict with professionals about my child.

- Strongly Agree
- Agree
- Disagree
- Strongly Disagree

I find that professionals involved with my child often don't believe me.

- Strongly Agree
- Agree
- Disagree
- Strongly Disagree

I find it easy to know which details about my child and family are appropriate to share with professionals involved with my child.

- Strongly Agree
- Agree
- Disagree
- Strongly Disagree

I find talking to professionals about my child causes me so much anxiety that I am unable to think clearly.

- Strongly Agree
- Agree
- Disagree
- Strongly Disagree

I find talking to professionals about my child causes me so much anxiety that I experience selective mutism or other forms of communication difficulty.

- Strongly Agree
- Agree
- Disagree
- Strongly Disagree

**Experiences with social services**

*This section is only displayed to individuals accessing the questionnaire from a United Kingdom IP address.*

Have any of the following experiences happened to you? Please answer yes or no.

My child or children have been assessed by social services as at risk of harm.

This means that an initial assessment of your child’s risk of harm would have been conducted after concerns about your child were reported to social services.

- Yes
- No

I have been called to a meeting with social services about my child.

- Yes
- No

Answer If Yes Is Selected

When I was called to the meeting with social services, I didn't understand what the purpose of the meeting was or what the legal implications of the meeting were.

- Strongly Agree
- Agree
- Disagree
- Strongly Disagree

My child or children have been placed on the child protection register.

- Yes
- No

Answer If Yes is Selected

What was the reason for them being placed on the child protection register?

|  |
| --- |

My child or children have been temporarily taken out of my care under a fostering order.

This means that your child or children would have been temporarily been placed in care.

- Yes
- No

Answer If Yes Is Selected

What was the reason for them being temporarily taken out of your care?

|  |
| --- |

My child or children have been permanently taken out of my care under an adoption order or a placement order.

This means that your child or children would have been permanently placed in care.

- Yes
- No

Answer If Yes Is Selected

What was the reason for them being permanently taken out of your care?

|  |
| --- |

I have been investigated for Munchausen Syndrome by Proxy or Fabricating Illness in my child or children.

- Yes
- No

Has social services ever said things about you that you felt were untrue or inaccurate?

- Yes
- No

Answer If Yes Is Selected

What was said that you felt was untrue or inaccurate?

|  |
| --- |

**Experience of motherhood**

I have found motherhood to be a rewarding experience.

- Strongly Agree
- Agree
- Disagree
- Strongly Disagree

I have found motherhood to be an isolating experience.

- Strongly Agree
- Agree
- Disagree
- Strongly Disagree

I am not afraid of others judging my parenting.

- Strongly Agree
- Agree
- Disagree
- Strongly Disagree

I am able to turn to others for support in parenting when I need it.

- Strongly Agree
- Agree
- Disagree
- Strongly Disagree

I have often felt I am not coping in being a mother.

- Strongly Agree
- Agree
- Disagree
- Strongly Disagree

I feel I should be offered extra support in my parenting, due to my autism spectrum condition.

- Strongly Agree
- Agree
- Disagree
- Strongly Disagree

What kind of extra support would you find useful? Please check all that apply.

- An autism advocate to accompany me to meetings with professionals about my children
- Respite care for my children or a carer's break
- Peer support from other mothers with ASC
- Assistance with the everyday tasks of parenting
- Other (please describe) ____________________

When I have asked for extra support from agencies in order to meet my needs as a parent, I have received the support I required.

- Strongly Agree
- Agree
- Disagree
- Strongly Disagree
- Not Applicable

**Disclosure of ASC**

How often do you disclose your autism spectrum condition to professionals when talking about your child?

- All of the Time
- Often
- Sometimes
- Rarely
- Never

If I were to disclose my diagnosis of ASC to a professional, I would worry about whether this person’s attitude towards me would change as a result of disclosure.

- Strongly Agree
- Agree
- Disagree
- Strongly Disagree

Answer If Strongly Agree Is Selected Or If Agree Is Selected

How might you think the person's attitude towards you might change?

|  |
| --- |

Do you ever encounter disbelief about your diagnosis of ASC after disclosing this diagnosis to a professional?

- All of the time
- Often
- Sometimes
- Rarely
- Never

Answer If Any Choice But Never Is Selected

Why have professionals disbelieved your diagnosis?

|  |
| --- |

What influences your decision whether or not to disclose your diagnosis to professionals?

|  |
| --- |

**Your Experiences**

What are some positive aspects of being a mother with an autism spectrum condition?

|  |
| --- |

What are the key issues you face as a mother with an autism spectrum condition?

|  |
| --- |
